# Supplementary material for: Competition between H4PteGlu and H2PtePAS Confers para-Aminosalicylic Acid Resistance in Mycobacterium tuberculosis
Source: Antibiotics (Basel). 2023 Dec 21;13(1):13. doi: 10.3390/antibiotics13010013 (PMC10812664; doi:10.3390/antibiotics13010013)
Supplement: Supplementary file 1 [file antibiotics-13-00013-s001.zip › antibiotics-2756637-supplementary.pdf]

Supplementary Table S1. Collection parameters of multiple reaction monitoring (MRM).

Bio-transformed Products	RT (min)	Ionization channel	Electrospray ionization
H_2PtePAS	3.193	331.0 -> 178.0	ESI+
H_2PtePAS-Glu	3.181	460.0 -> 178.0	ESI+

Supplementary Table S2. Mutation analysis of *ribD* in ThyA or DfrA deficient *M. tuberculosis* isolates.

genome_ID	*thyA*_CDS	*dfrA*_CDS	*ribD*_promoter	*ribD*_CDS	Reference
ERR502929	deletion	deletion	WT	WT	
ERR751453	deletion	deletion	WT	WT	[22]
ERR775373	deletion	deletion	WT	WT	
ERR779910	deletion	deletion	WT	WT	
SRR16588749	deletion	deletion	WT	WT	[11]
SRR1952721	deletion	deletion	WT	WT	
SRR2328057	deletion	deletion	WT	WT	[27]
SRR2333215	deletion	deletion	WT	WT	
GCA_000177835.2	Leu63fs	WT	WT	WT	
GCA_000652475.1	Thr22fs	WT	WT	WT	
GCA_000657935.1	Gln165*	WT	WT	WT	
GCA_000657955.1	Gln165*	WT	WT	WT	
GCA_000657995.1	Gln165*	WT	WT	WT	
GCA_000658015.1	Gln165*	WT	WT	WT	
GCA_000669315.1	Gln165*	WT	WT	WT	
GCA_000669395.1	Gln165*	WT	WT	WT	
GCA_000669555.1	Gln165*	WT	WT	WT	
GCA_000673575.1	Gln165*	WT	WT	WT	
GCA_000673735.1	Gln165*	WT	WT	WT	
GCA_000673755.1	Glu139*	WT	WT	WT	NCBI database
GCA_000674395.1	Trp80*	WT	WT	WT	
GCA_000674475.1	Gln165*	WT	WT	WT	
GCA_000674535.1	Arg99*	WT	WT	WT	
GCA_000674575.1	Gln165*	WT	WT	WT	
GCA_000677115.1	Trp133*	WT	WT	WT	
GCA_000678495.1	Gln111*	WT	WT	WT	
GCA_000679475.1	Leu63fs	WT	WT	WT	
GCA_000804245.1	Glu139*	WT	WT	WT	
GCA_003392965.1	Ile69fs	WT	WT	WT	
GCA_014900815.1	Tyr4fs	WT	WT	WT	
GCA_021026275.1	Ser180fs	WT	WT	WT	

Fs, frameshift; *, stop transcription

Supplementary Table S3. Plasmids, strains, and primers used in this study.

Category	Name	Characterization/Sequence (5'-3')	Source
**Plasmids**			
	pMAL-c2X	*E. coli* expression plasmid under control of Tac promoter with N-terminal MBP tag linked with a factor Xa cleavage site, Ap^R	New England BioLabs
	pMAL-c2X::*folC*	pMAL-c2X with *M. tuberculosis* H37Rv *folC* inserted downstream of the Tac promoter, Ap^R	This study
	pET28a	*E. coli* expression plasmid under control of T7 promoter with N-terminal His tag, Km^R	Novagen
	pET28a::*folP1*	pET28a with *M. tuberculosis* H37Rv *folP1* inserted downstream of the T7 promoter, Km^R	This study
	pMV261	Non-integrated shuttle plasmid under control of *hsp60* promoter, Km^R	Prof. William R Jacobs Jr, Albert Einsterin College of Medicine
	pMV261::*thyA*	pMV261 with *M. tuberculosis* H37Rv *thyA* inserted downstream of the *hsp60* promoter, Km^R	This study
	pMV261::*thyX*	pMV261 with *M. tuberculosis* H37Rv *thyX* inserted downstream of the *hsp60* promoter, Km^R	This study
	pMV261::*folP1*	pMV261 with *M. tuberculosis* H37Rv *folP1* inserted downstream of the *hsp60* promoter, Km^R	This study
	pMV261::*folC*	pMV261 with *M. tuberculosis* H37Rv *folC* inserted downstream of the *hsp60* promoter, Km^R	This study
	pMV261::*dfrA*	pMV261 with *M. tuberculosis* H37Rv *dfrA* inserted downstream of the *hsp60* promoter, Km^R	This study
	p0004s	Homologous arms construction plasmid, $HygB^R$	Prof. William R Jacobs Jr, Albert Einsterin College of Medicine
	phAE159	Phage transduction plasmid, Ap^R	Prof. William R Jacobs Jr, Albert Einsterin College of Medicine
**Strains**			
	E. coli BL21 (DE3)	Host for protein expression	New England BioLabs
	BL21 (DE3) pMAL-c2X::*folC*	*E. coli* BL21 (DE3) transformed with pMAL-c2X::*folC*	This study

	BL21 (DE3) pET28a::*folP1*	*E. coli* BL21 (DE3) transformed with pET28a::*folP1*	This study
	E. coli HB101	Host for plasmid amplification	Prof. William R Jacobs Jr, Albert Einstein College of Medicine
	M. smegmatis mc^{2}155	High-efficiency transformation strain	Prof. William R Jacobs Jr, Albert Einstein College of Medicine
	H37Ra	*M. tuberculosis* H37Ra avirulent strains	Prof. William R Jacobs Jr, Albert Einstein College of Medicine
	H37Ra Δ*thyA*	*M. tuberculosis* H37Ra with *thyA* gene deletion	This study
	H37Ra pMV261	*M. tuberculosis* H37Ra transformed with pMV261	This study
	H37Ra Δ*thyA* pMV261	*M. tuberculosis* H37Ra Δ*thyA* transformed with pMV261	This study
	H37Ra Δ*thyA* pMV261::*thyA*	*M. tuberculosis* H37Ra Δ*thyA* transformed with pMV261::*thyA*	This study
	H37Ra Δ*thyA* pMV261::*thyX*	*M. tuberculosis* H37Ra Δ*thyA* transformed with pMV261::*thyX*	This study
	H37Ra Δ*thyA* pMV261::*folP1*	*M. tuberculosis* H37Ra Δ*thyA* transformed with pMV261::*folP1*	This study
	H37Ra Δ*thyA* pMV261::*folC*	*M. tuberculosis* H37Ra Δ*thyA* transformed with pMV261::*folC*	This study
	H37Ra Δ*thyA* pMV261::*dfrA*	*M. tuberculosis* H37Ra Δ*thyA* transformed with pMV261::*dfrA*	This study
	H37Ra pMV261::*thyA*	*M. tuberculosis* H37Ra transformed with pMV261::*thyA*	This study
	H37Ra pMV261::*thyX*	*M. tuberculosis* H37Ra transformed with pMV261::*thyX*	This study
	H37Ra pMV261::*folP1*	*M. tuberculosis* H37Ra transformed with pMV261::*folP1*	This study
	H37Ra pMV261::*folC*	*M. tuberculosis* H37Ra transformed with pMV261::*folC*	This study
	H37Ra pMV261::*dfrA*	*M. tuberculosis* H37Ra transformed with pMV261::*dfrA*	This study
	F461	Clinical isolate of *M. tuberculosis* H37Rv with mutation *thyA*(^{235}Arg→Pro)	Chongqing Public Health Medical Center
	F461 pMV261::*folC*	*thyA*(^{235}Arg→Pro) transformed with pMV261::*folC*	This study
**Primers**			

	folC-BamHI-F	TTAAGGATCCATGAATTCGACGAATTCCGG	This study
	folC-HindIII-R	TTACAAGCTTTTATTGCGGATCACGACC	This study
	folP1-EcoRI-F	ATAGAATTCATGAGTCCGGCGCCCGTGCA	This study
	folP1-HindIII-R	ATAAAGCTTTTAGCCATCGCGTTCTATCCTTTCC	This study
	dfrA-BamHI-F	TTAAGGATCCATGGTGGGGCTGATCTGGGCT	This study
	dfrA-HindIII-R	TAATAAGCTTTTATGAGCGGTGGTAGCTGTAC	This study
	thyA-BamHI-F	TTAAGGATCCATGACGCCATACGAGGACCTGCTGC	This study
	thyA-HindIII-R	TACTAAGCTTTTATACCGCGACTGGAGCTTTGATCG	This study
	thyX-BamHI-F	TTAAGGATCCATGGCCGAGACCGCGCCGCTG	This study
	thyX-HindIII-R	TACTAAGCTTTTAGGCTTCGGTCGCCAACGGGCT	This study
	pMV261-JDFP	GTGGCAGCGAGGACAACTTG	This study
	pMV261-JDRP	GATGCCTGGCAGTCGATCGTAC	This study
	thyAko-LFP	TTTTTTTTCCATAAATTGGCCGGGTAGCACTCGATGCGGTGCG	This study
	thyAko-LRP	TTTTTTTTCCATTTCTTGGTCGAGCACGAAGCGCAGCAGGTCCT	This study
	thyAko-RFP	TTTTTTTTCCATAGATTGGATCCGCATCCGGCGATCAAAGCT	This study
	thyAko-RRP	TTTTTTTTCCATCTTTTGGGTAATACAAGTGCCGGCGTTTGGCC	This study
	thyA-IDF	ATCGGGATCGACGTCGCGGCGCCT	This study
	thyA-IDR	AGATCACCCCGGGTGGACACGGCGAT	This study

Supplementary Figures and Figure Legends

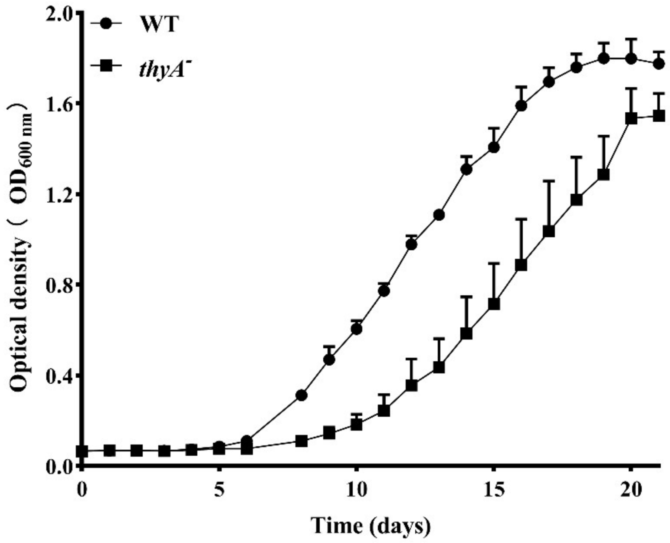

Figure S1. Growth curves of H37Ra (WT) and H37Ra Δ*thyA* (*thyA*-) in liquid culture at 37°C. The OD_{600} was measured by using a SynergyH1 Hybrid reader (BioTek, USA). Data represent the means of three biological replicates, and error bars denote the standard deviations.

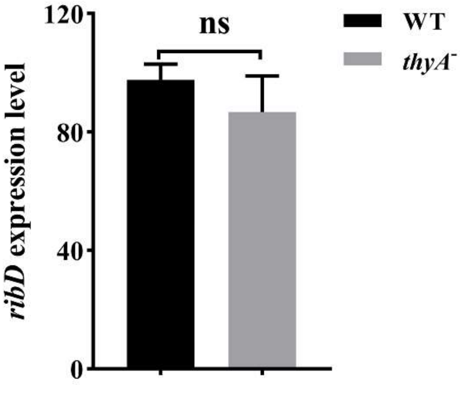

Figure S2. Comparison of the transcriptional level of the *ribD* gene during the exponential phase in H37Ra (WT) and H37Ra Δ*thyA* (*thyA*-) by RNA-seq. ns, no significance.
